# Chest X-Ray Not Routinely Indicated Prior to the YEARS Algorithm in the Diagnostic Management of Suspected Pulmonary Embolism

**DOI:** 10.1055/s-0038-1676812

**Published:** 2019-01-08

**Authors:** Liselotte M. van der Pol, Cecile Tromeur, Laura M. Faber, Tom van der Hulle, Lucia J. M. Kroft, Albert T. A. Mairuhu, Albert de Roos, Menno V. Huisman, Frederikus A. Klok

**Affiliations:** 1 Department of Medicine - Thrombosis and Hemostasis, Leiden University Medical Center, Leiden, The Netherlands; 2 Department of Internal Medicine, Haga Teaching Hospital, The Hague, The Netherlands; 3 Groupe d'Etude de la Thrombose de Bretagne Occidentale, Department of Internal Medicine and Chest Diseases, University of Brest, CHRU Brest, Brest, France; 4 Department of Internal Medicine, Red Cross Hospital, Beverwijk, The Netherlands; 5 Department of Radiology, Leiden University Medical Center, Leiden, The Netherlands

**Keywords:** pulmonary embolism, diagnosis, chest X-ray, clinical decision rule, D-dimer

## Abstract

**Background** The YEARS algorithm was designed to simplify the diagnostic process of suspected pulmonary embolism (PE) and to reduce the number of required computed tomography pulmonary angiography (CTPA) scans. Chest X-ray (CXR) is often used as initial imaging test in patients suspected for PE.

**Aim** To determine if CXR results differ between patients with confirmed PE and with PE ruled out, and to investigate whether CXR provides incremental diagnostic value to the YEARS criteria that is used for selecting patients with CTPA indication.

**Methods** This post-hoc analysis concerned 1,473 consecutive patients with suspected PE who were managed according to YEARS and were subjected to CXR as part of routine care. The prevalence and likelihood ratios of seven main CXR findings for a final diagnosis of PE were calculated.

**Results** A total of 214 patients were diagnosed with PE at baseline (15%). Abnormal CXR occurred more often in patients with confirmed PE (36%, 77/214) than in patients without PE (26%; 327/1,259), for an odds ratio of 1.60 (95% confidence interval: 1.18–2.18). Only the unexpected finding of a (rib)fracture or pneumothorax, present in as few as six patients (0.4%), significantly lowered the post-test probability of PE to an extent that CTPA could have been avoided.

**Conclusion** The incremental diagnostic value of CXR to the YEARS algorithm to rule out PE was limited. CXR was more frequently abnormal in patients with PE than in those in whom PE was ruled out. These data do not support to perform CXR routinely in all patients with suspected PE, prior to CTPA imaging.

## Introduction

The diagnostic management of suspected acute pulmonary embolism (PE) is challenging due to its nonspecific signs and symptoms. The clinical suspicion of PE must therefore be followed by objective testing. Current guidelines recommend applying clinical decision rules to categorize patients in accordance with their pretest probability of PE. 1 2 In case of non-high probability of PE, D-dimer testing is warranted because PE can be safely ruled out if the D-dimer test result is normal. In case of abnormal D-dimer or high clinical probability, computed tomography pulmonary angiography (CTPA) should be performed. 1 2 3 In recent years, attempts have been made to increase the number of patients in whom imaging is not required to rule out PE, for instance by introduction of an age-dependent D-dimer threshold. A recently published strategy is the simple and straightforward YEARS algorithm that includes simultaneous D-dimer and clinical pretest probability assessment and the application of a pretest probability D-dimer threshold. 4 5 The YEARS algorithm was shown to safely rule out acute PE (failure rate of the overall algorithm: 0.61%, 95% confidence interval [CI]: 0.36–0.96) and reduce the need for CTPA examinations by 14% compared with the conventional diagnostic strategy. 5

Chest X-ray (CXR) is a commonly performed test in the initial evaluation of suspected cardiopulmonary disease and has the advantage of being associated with lower radiation exposure than CTPA. 6 Since 40 to 88% of patients with PE have mostly nonspecific abnormal CXR findings, CXR cannot be used to confirm and/or exclude the diagnosis of PE, 6 7 8 although it may indicate other cardiopulmonary conditions. 6 Most prevalent abnormal CXR findings in PE patients are cardiomegaly, atelectasis, elevated hemi diaphragm, pleural effusion, pulmonary infarction, and parenchymal areas of increased opacity. 6 7 9 10 11 12 Interestingly, the NICE guideline recommends to start the diagnostic management of patients with suspected PE with a CXR to exclude other conditions than acute PE. 13 Strong evidence supporting this recommendation is lacking. The aim of the current analysis was to investigate whether a CXR provides incremental diagnostic value to the YEARS algorithm in the diagnostic workup of suspected acute PE.

## Methods

### Study Population

**Fig. 1:**
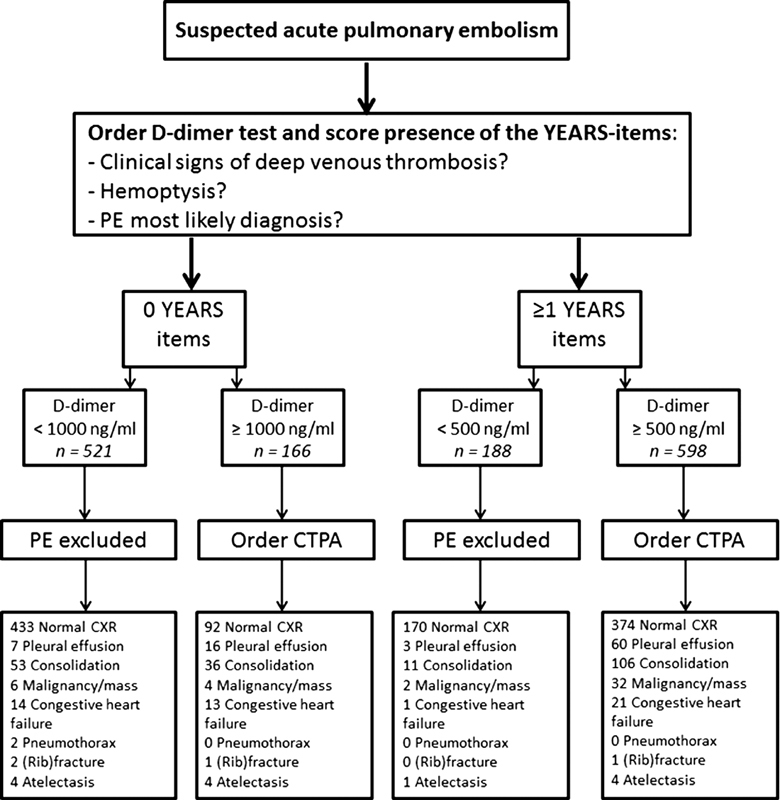
The YEARS algorithm.

For this post hoc analysis, 1,711 consecutive patients with suspected PE from the YEARS study from three Dutch hospitals, in which a CXR was performed as part of routine clinical care, were evaluated. All patients were managed according to the YEARS algorithm ( Fig. 1 ). 5 Exclusion criteria for the YEARS study were treatment with anticoagulants in therapeutic doses initiated ≥ 24 hours before inclusion, life expectancy less than 3 months, pregnancy, or allergy to intravenous contrast agents. 5 Patients with confirmed PE were treated with anticoagulants according to international guidelines. Follow-up consisted of a scheduled outpatient clinic visit or telephone interview after 3 months.

### Chest X-Rays

All patients included in this analysis underwent a CXR in the diagnostic workup for suspected PE before they were referred for CTPA. The results of the CXR were reported by the local attending radiologist, who was either a certified radiologist or a resident under supervision of a certified radiologist. For this analysis, CXRs were classified as normal or abnormal. The following abnormalities were recorded: pleural effusion, consolidation, malignancy/mass, congestive heart failure, pneumothorax, (rib) fracture, and atelectasis.

### Aims and Endpoints of this Analysis

The aim of this analysis was to determine the prevalence of CXR abnormalities among patients with suspected PE and to evaluate if CXR results differ in patients with confirmed PE versus patients with PE ruled out. Further aims were to investigate the potential incremental value of performing a CXR routinely in all patients with suspected PE, that is, whether the posttest probability of PE after certain CXR findings would allow for changing the decision to perform CTPA as indicated by YEARS.

The endpoints of this analysis included the odds ratios (OR; with 95% CI) between the rate of abnormalities on CXR for patients with confirmed PE versus patients with PE ruled out, and for patients with an indication for CTPA according to the YEARS algorithm versus patients without CTPA indication. Furthermore, we assessed the positive and negative likelihood ratios (LRs) for the specific predefined CXR abnormalities mentioned earlier, to calculate the posttest probability for each abnormality.

### Statistical Analysis

To compare the rate of abnormalities on CXR for patients with PE versus those without PE, an OR with corresponding 95% CI was calculated. To evaluate whether the posttest probability of PE changed after the CXR result, positive and negative LR with 95% CIs were calculated for each different abnormality on CXR and for a normal CXR. The pretest probability was dependent on the PE prevalence, which was calculated in all patients and in patients who were referred for CTPA according to the YEARS algorithm. All analyses were performed using SPSS, version 23.0, Chicago, Illinois, United States.

## Results

### Patient Characteristics

From the 1,711 eligible patients, CXR was not performed in 238 patients for unknown reasons. After excluding these patients, 1,473 were left for analysis. Their mean age was 54 years, 62% were female, 14% of patients had COPD, 2% had chronic heart failure, and 9% had an active malignancy. Dyspnea was present in 71% of these patients, 40% presented with coughing and 74% with thoracic pain ( Table 1 ). The patients who were managed without CXR had numerical but not significant less comorbidities than the included patients: 6.3% of these patients were known with COPD and 1.1% of these patients had a history of heart failure, the majority were women (72%) and the mean age was 53 years. Following the YEARS algorithm, CTPA was indicated to rule out or confirm the diagnosis of PE in 763 (52%) of all patients. PE was diagnosed in 214 patients at baseline for a prevalence of 15%. Prevalence of PE among the 238 excluded patients without CXR was 17%.

**Table 1:** Baseline characteristics

	All patients ( *n* = 1,473)
Mean age, y (SD)	54.4 (18.6)
Female sex, *n* (%)	922 (62.6)
Pulmonary embolism, *n* (%)	214 (14.5)
CTPA indicated following YEARS, *n* (%)	763 (51.8)
Prior VTE, *n* (%)	146 (9.9)
COPD, *n* (%)	208 (14.1)
Heart failure, *n* (%)	30 (2.0)
Malignancy, *n* (%)	133 (9.0)
Immobilization or recent surgery, *n* (%)	159 (10.8)
Use of estrogen in women, *n* (%)	131 (14.2)
Smoking, *n* (%)	250 (23.8)
Symptoms, n (%)	
Dyspnea	1,045 (70.9)
Coughing	579 (39.3)
Thoracic pain	1,086 (73.7)
Palpitations	115 (7.8)
Fever (>38.5°C)	47 (3.2)

Abbreviations: COPD, chronic obstructive pulmonary disease; CTPA, computed tomography pulmonary angiography; VTE, venous thromboembolism.

### CXR Results in All Patients

**Fig. 2:**
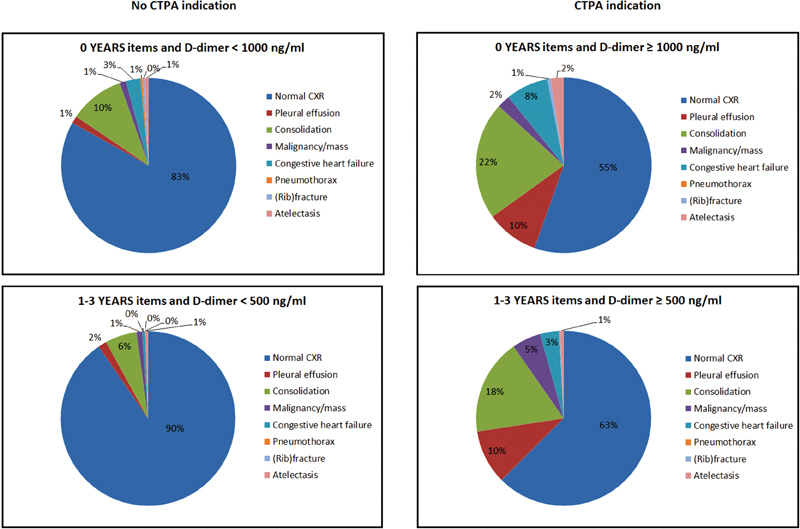
CXR findings per YEARS group.

The majority of patients had a normal CXR (73%). Abnormal CXR was more frequent among patients with confirmed PE (36%) than in patients without PE (26%; Table 2 ) for an OR of 1.60 (95% CI: 1.18–2.18). Consolidation was the most frequent abnormality which was present in 23% of patients with PE versus 13% of patients without PE for an OR of 2.08 (95% CI: 1.45–2.99). Other CXR abnormalities were quite similar between the group of patients with and without PE ( Table 2 ). The distribution of specific CXR results in different patient groups is illustrated in Fig. 2 .

**Table 2:** Overview of CXR findings in different patient groups

Result of CXR	All patients ( *n* = 1,473)	PE ( *n* = 214)	No PE ( *n* = 1,259)	CTPA indicated ( *n* = 763)
Normal CXR, *n* (%)	1,069 (72.6)	137 (64.0)	932 (74.0)	465 (60.9)
Pleural effusion, *n* (%)	86 (5.8)	14 (6.5)	72 (5.7)	76 (10.0)
Consolidation, *n* (%)	206 (14.0)	49 (22.9)	157 (12.5)	142 (18.6)
Malignancy/mass, *n* (%)	44 (3.0)	6 (2.8)	38 (3.0)	36 (4.7)
Congestive heart failure, *n* (%)	49 (3.3)	7 (3.3)	42 (3.3)	34 (4.5)
Pneumothorax, *n* (%)	2 (0.1)	0 (0.0)	2 (0.2)	0 (0.0)
(Rib) fracture, *n* (%)	4 (0.3)	0 (0.0)	4 (0.3)	2 (0.3)
Atelectasis, *n* (%)	13 (0.9)	1 (0.5)	12 (1.0)	8 (1.0)

Abbreviations: CTPA, computed tomography pulmonary angiography; CXR, chest X-ray; PE, pulmonary embolism.

### CXR Results in Patients with an Indication for CTPA

From the 763 patients with an indication for CTPA according to the YEARS algorithm, the CXR was normal in 465 patients (61%) compared with 604 of the 710 patients (85%) in whom CTPA was not indicated for an OR of 3.65 (95% CI: 2.84–4.70). Consolidation was the most frequent abnormality found on CXR in all four different YEARS groups ( Fig. 2 ).

### Pre- and Posttest Probability after CXR

Table 3 illustrates the different LRs with 95% CI for all predefined CXR abnormalities. Pneumothorax and rib fracture were rare, with prevalence of only 0.1 and 0.3%, respectively. For the overall population, only these two rare findings significantly lowered the posttest probability of PE with a positive LR of 0.00 ( Table 3 ). Most of the other LRs were around 1.00, indicating that the result of the CXR did not change the posttest probability of a PE diagnosis. For patients with an indication for CTPA, only the CXR finding of a rib fracture, which was present in two patients, lowered the posttest probability to such an extent that CTPA could have been avoided ( Table 3 ). Atelectasis on the CXR in patients with an indication for CTPA lowered the posttest probability on PE with a LR of 0.37, although with a broad 95% CI (0.05–3.0), consistent with an 8% (1/13) prevalence of PE in patients with atelectasis.

**Table 3:** Overview of LRs and CXR results in two groups; all patients and patients in whom CTPA was indicated according to the YEARS algorithm

	All patients ( *n* = 1,473)		Patients in whom CTPA was indicated according to the YEARS algorithm ( *n* = 763)	
Results CXR	Positive LR (95% CI)	Negative LR (95% CI)	Positive LR (95% CI)	Negative LR (95% CI)
Normal CXR	0.86 (0.78–0.96)	1.4 (1.1–1.7)	1.1 (0.95–1.2)	0.89 (0.73–1.1)
Pleural effusion	1.1 (0.66–2.0)	0.99 (0.95–1.0)	0.58 (0.33–1.0)	1.1 (1.0–1.1)
Consolidation	1.8 (1.4–2.4)	0.88 (0.82–0.95)	1.4 (0.99–1.8)	0.93 (0.86–1.0)
Malignancy/mass	0.93 (0.40–2.2)	1.0 (0.98–1.0)	0.51 (0.22–1.2)	1.0 (1.0–1.1)
Congestive heart failure	0.98 (0.45–2.2)	1.0 (0.97–1.0)	0.67 (0.29–1.5)	1.0 (0.99–1.1)
Pneumothorax	0.00	1.0 (0.99–1.0)	n.a.	n.a.
(Rib) fracture	0.00	1.0 (0.99–1.0)	0.00	1.0 (0.99–1.0)
Atelectasis	0.49 (0.06–3.8)	1.0 (0.99–1.0)	0.37 (0.05–3.0)	1.0 (0.99–1.0)

Abbreviations: CI, confidence interval; CTPA, computed tomography pulmonary angiography; CXR, chest X-ray; LR, likelihood ratio; n.a., not applicable; PE, pulmonary embolism.

Example: Assuming that the pretest probability of PE is 28% in a certain patient with suspected PE and an indication for CTPA according to YEARS, the posttest probability of PE in case of a normal CXR result would be 28% × 1.1 = 31%. The posttest probability of PE in this patient with any abnormality on CXR would be 28% × 0.89 = 25%.

## Discussion

In our cohort of patients with suspected PE, CXR was more frequently abnormal in patients who were diagnosed with PE than in those in whom PE was ruled out. The posttest probability of PE was only relevantly changed in patients with a (rib) fracture and/or a pneumothorax, which were rare findings. The incremental diagnostic value of CXR to the YEARS algorithm to rule out PE was therefore limited.

Previous studies have shown conflicting results for abnormalities on CXR observed in patients with PE. Two studies reported cardiomegaly as most common abnormality with a prevalence of 38% (19/50 patients) and 27% (622/2,315 patients), respectively. 6 10 Robin et al found interstitial lung disease or consolidation as most prevalent abnormality (28%) 11 and two other retrospective studies, of which one was the PIOPED study, reported atelectasis/parenchymal areas with increased opacity as most common abnormality with a prevalence of 68%. 7 12 This heterogeneity in CXR findings demonstrates that a suspicion of acute PE may cause different nonspecific abnormalities on CXR. Considering this, the diagnostic value of CXR for the diagnosis of PE is therefore poor.

In the past, CXR was used as standard imaging test in the approach of patients with suspected PE to find alternative diagnosis and as a useful tool for the interpretation of the ventilation/perfusion scan (V/Q scan). 7 11 12 Nowadays, CTPA is the first-choice imaging test in the diagnostic workup for patients with suspected PE due to the ability to directly visualize emboli, as well as alternative diagnosis. CXR is not needed for interpretation of the CTPA. Reasons why CXR is often used in clinical practice are its wide availability, the fast execution, the low radiation exposure compared with CTPA or VQ scan, and the low costs. 14 Patients without an indication for CTPA, and thus a lower probability on PE, had more often a normal CXR in our cohort than patients referred for CTPA. However, normal CXR as well as abnormal CXR could not distinguish patients with from those without CTPA indication, nor could CXR distinguish between patients with or without PE.

CXR may have two different roles in the diagnostic workup of patients with suspected PE. First of all, CXR is an important diagnostic modality at the emergency department for the initial assessment of patients with respiratory and/or chest symptoms. The result of the CXR could lead to change suspected PE to another diagnosis or to moving PE higher up in the differential diagnosis. Moreover, the results of CXR, which were likely available for some of the patients when the physician completed the YEARS algorithm, could have led to assigning less or more YEARS items to the patient. This may have influenced the final YEARS classification and associated D-dimer threshold. For those reasons, CXR could therefore have contributed to the efficiency of the YEARS algorithm. 5 Second, the CXR may be used as a routine test to exclude alternative diagnosis before performing a CTPA.

A limitation of this study is that because of the retrospective design and the lack of detailed information on the differential diagnosis of each individual patient, we were not able to explore the reason why the CXR was ordered, especially because CXR is no longer recommended nor considered among the useful tools for this specific clinical setting in recent guidelines. Also, for unknown reasons, not all patients in our cohort were referred for CXR which may have caused selection bias. Even so, CXR results hardly influenced the posttest probability of PE in any of the YEARS categories. Therefore, our data do not support routine use of CXR in all patients before CTPA. Also, for unknown reasons, not all patients in our cohort were referred for CXR which may have caused selection bias.

In conclusion, CXR shows nonspecific abnormalities in patients with confirmed PE more frequently than in patents with PE ruled out. Only the rare findings of a (rib) fracture or pneumothorax significantly lowered the posttest probability to such an extent that CTPA could have been avoided according to the YEARS algorithm. Although CXR may play an important role in the initial diagnostic management in patients with suspected PE, our data do not support routine CXR in all patients with suspected PE, especially not in patients with an established indication for CTPA by YEARS.
